# Obstacle Avoidance, Visual Detection Performance, and Eye-Scanning Behavior of Glaucoma Patients in a Driving Simulator: A Preliminary Study

**DOI:** 10.1371/journal.pone.0077294

**Published:** 2013-10-16

**Authors:** Rocío Prado Vega, Peter M. van Leeuwen, Elizabeth Rendón Vélez, Hans G. Lemij, Joost C. F. de Winter

**Affiliations:** 1 Department of BioMechanical Engineering, Delft University of Technology, Delft, The Netherlands; 2 Rotterdam Ophthalmic Institute (ROI), Rotterdam, The Netherlands; 3 Rotterdam Eye Hospital (REH), Rotterdam, The Netherlands; Bascom Palmer Eye Institute, University of Miami School of Medicine;, United States of America

## Abstract

The objective of this study was to evaluate differences in driving performance, visual detection performance, and eye-scanning behavior between glaucoma patients and control participants without glaucoma. Glaucoma patients (n = 23) and control participants (n = 12) completed four 5-min driving sessions in a simulator. The participants were instructed to maintain the car in the right lane of a two-lane highway while their speed was automatically maintained at 100 km/h. Additional tasks per session were: Session 1: none, Session 2: verbalization of projected letters, Session 3: avoidance of static obstacles, and Session 4: combined letter verbalization and avoidance of static obstacles. Eye-scanning behavior was recorded with an eye-tracker. Results showed no statistically significant differences between patients and control participants for lane keeping, obstacle avoidance, and eye-scanning behavior. Steering activity, number of missed letters, and letter reaction time were significantly higher for glaucoma patients than for control participants. In conclusion, glaucoma patients were able to avoid objects and maintain a nominal lane keeping performance, but applied more steering input than control participants, and were more likely than control participants to miss peripherally projected stimuli. The eye-tracking results suggest that glaucoma patients did not use extra visual search to compensate for their visual field loss. Limitations of the study, such as small sample size, are discussed.

## Introduction

Glaucoma can produce severe visual impairment and is the second leading cause of blindness [1]. It has been estimated that 60 million people are currently afflicted with glaucoma, and this number is expected to increase as the population ages [2]. Many glaucoma patients tend to self-regulate their driving activity by avoiding difficult situations such as driving in the dark and rain, or during peak hours [3,4]. However, some glaucoma patients with advanced visual field loss continue to drive [4] and may endanger themselves and others. Several studies have shown that glaucoma patients are overrepresented in self-reported and police-registered motor vehicle collisions [5-9].

Results from an on-road driving test showed that glaucoma patients (n = 20) performed common driving maneuvers (e.g., entering traffic, turning, negotiating intersections, and parking) as adequately as age-matched controls (n = 20) [10]. However, the study also showed that a driving instructor intervened more often when glaucoma patients drove than when control subjects drove (60% vs. 20% of participants, respectively). The interventions were related to potentially unsafe driving behaviors, such as failure to see and yield to a pedestrian or an oncoming vehicle, and failure to see and stop at a stop sign. In another on-road study, it was found that glaucoma patients with more binocular field loss performed worse at dynamic tasks such as taking turns and lane changing [11]. 

Recently, research interest has increased in determining valid fitness-to-drive criteria for glaucoma patients [12]. On-road tests are traditionally regarded as the gold standard in driving assessment [13]. However, human examiners have limited inter-rater reliability [14-16] and even if examiners were always consistent and in agreement with all other examiners, there would still be limitations in the reliability and validity of on-road testing, due to random traffic and weather conditions, as well as local differences in road infrastructure. In a recent review, Medeiros et al. [12] proposed driving simulators as a tool for evaluating driving performance of glaucoma patients. Driving simulators may be able to predict on-road driving safety because they provide the visual and auditory sensations that mimic those encountered in real car driving, while providing high controllability and repeatability. 

A number of previous studies have evaluated driving performance of glaucoma patients in a driving simulator. Szlyk et al. [17] compared 25 patients having mild to moderate glaucoma with 29 normally-sighted control participants of equivalent age. The patient and control groups performed indistinguishably on most of the driving simulator indices (e.g., mean speed, number of lane boundary crossings, number of simulator accidents), but a substantial difference was found in brake response times, where patients pressed the brake pedal more rapidly after the presentation of a stop sign (2.19 s for patients vs. 4.81 s for control participants, p < 0.001). The authors suggested that this effect was not caused by different visual abilities of the two groups per se, but by the patients being vigilant and trying to react immediately, whereas the control group adopted a longer wait time, reaching a point closer to the intersection. In a later study [9], 40 glaucoma patients with a wide range of visual field loss were compared with 17 normally-sighted control participants as to their performance on an 8-min evaluation course, while being instructed to drive as they normally would and to obey traffic rules. The results showed that the glaucoma group had a higher incidence of accidents in the simulator. More recently, Rosen et al. [8] tested 45 glaucoma patients and 76 participants without glaucoma in a driving simulator. Compared to the control group, the glaucoma patients demonstrated a longer time delay during car following, and had a longer reaction time and missed more targets on a detection task. This detection task involved targets that were randomly presented in the right or left peripheral visual field, while the participant performed a task requiring central fixation (car following with variable speed of the lead vehicle). In the study by Rosen et al., 93% of the glaucoma patients had mild to moderate glaucoma. The authors stated that further research should be performed with patients having severe glaucoma, and that it is important to clarify the relationship between depth and location of visual field defects in simulators.

The aim of the present study was to evaluate differences between patients with mild to severe glaucoma and control participants regarding their driving performance, visual detection performance, and eye-scanning behavior in a simulator. In agreement with Haymes et al. [10], we speculated that glaucoma patients are able to adequately keep the car on the road and avoid collisions, but are less likely to detect unexpected events in the periphery. A test was designed to evaluate lane keeping and obstacle avoidance, as well as detection of projected letters. The projected letters were regarded as surrogates for unexpected events occurring in the environment. In addition, the relationship between task performance and visual field loss was investigated. We speculated that more severe visual field defects would result in a worse letter detection performance. 

Previous research suggests that persons with hemianopsia or with an artificially restricted field of view adjust their head movements and eye-scanning behavior as a compensation technique for the loss of visual information [18-21]. A driving simulator study by Coeckelbergh et al. [22] reported that participants with peripheral visual field defects who passed an on-road driving test made more head movements when approaching an intersection in the driving simulator than those who failed the on-road test. However, no statistically significant correlations were found between visual field loss and head movements. In a recent exploratory study using video-based hazard-perception tasks, Crabb et al. [23] reported that glaucoma patients made more eye movements (i.e., shorter fixations, and more saccades and fixations per second, but with equivalent saccade amplitudes) than control participants. The authors interpreted that patients, unconsciously or consciously, make these eye movements as a compensation for their restricted field of view. A preliminary driving simulator study by Lockhart et al. [24] found no statistically significant differences in head position variability and number of eye fixations to areas of interest (i.e., mirrors, dashboard, etc.) between drivers with peripheral visual field loss and drivers with normal vision. In our study, we explored patients’ eye-scanning behavior to investigate whether visual compensation occurred in glaucoma patients during driving.

## Materials and Methods

### Ethics statement

The research adhered to the tenets of the Declaration of Helsinki. This study was approved by the Medical Ethics Committee of the Erasmus Medical Centre, Rotterdam and the Human Research Ethics Committee of the Delft University of Technology. All individuals gave their written informed consent.

### Participants

Twenty-five glaucoma patients with Primary Open Angle Glaucoma (POAG) and 14 control participants without glaucoma were recruited from the Rotterdam Ophthalmic Institute (ROI) from an ongoing longitudinal follow-up study into glaucoma imaging.

Participants were measured with standard automated perimetry (SAP) by means of the Humphrey Field Analyzer II (Carl Zeiss Meditec, model number 750), with the 24-2 Swedish Interactive Threshold Algorithm (SITA; Software Version: 4.2.2). The exclusion criteria for all participants were: (1) having a best-corrected visual acuity worse than 6/12 on a Snellen Chart in at least one eye (2), having a refractive error outside the -10.0 to +5.0 diopters range (3), having had cataract surgery in the previous 12 months before the experiment (4), having had previous refractive or vitreoretinal surgery (5), presenting evidence of diabetic retinopathy, macular edema, or other vitreoretinal disease (6), having had previous keratoplastic surgery. There were no age cut-offs during recruitment. We attempted to invite patients and controls of corresponding ages, with the aim to keep the mean age of both groups approximately equal.

Glaucoma patients had a confirmed diagnosis based on optic nerve damage and a visual field loss according to the following measures: (1) Glaucoma Hemifield Test (GHT) result outside normal limits, and (2) 3 or more adjacent points depressed at p < 0.05 or 1 or more points at p < 0.01 in the total deviation plot. Patients with an eye disease other than glaucoma that might affect the visual field (including greater than mild cataract) or with secondary glaucoma were not included. In order to test various types of visual field loss, the patients were selected based on their scotoma pattern as follows: (1) defects in the left eye only (n = 4), (2) defects in the right eye only (n = 7), (3) defects in alternated regions (defects in the upper field of one of the eyes and the lower field of the fellow eye) (n = 5), (4) defects in the upper visual field of both eyes (n = 6), and (5) defects in the lower visual field of both eyes (n = 3). The control participants had a visual field within normal limits in the GHT and no history of ocular disease. 

### Procedures and driving instructions

Prior to the simulator test, participants filled out a 37-item multiple-choice questionnaire in Dutch language. This questionnaire consisted of 8 general items (e.g., age, gender, medication use, estimated health, worry about sight), 5 items about difficulties with daily activities, 10 items about driving history and self-rated driving performance (e.g., mileage, number of accidents, number of fines, driving skills), 12 items about violations and errors while driving, 1 item about preferred driving modality, and 1 item about experience with cruise control. The items were based on the Driving Habits Questionnaire [25], the National Eye Institute-Visual Functioning Questionnaire (NEI-VFQ) Driving Subscale [26], and the Manchester Driver Behaviour Questionnaire (DBQ) [27,28]. 

Next, the participants performed reaction time tests on a desktop computer, and received a video instruction of 3.5 minutes. The video described general indications for controlling the car and the tasks per session.

Participants then seated inside the driving cabin, and put on the seat belt in order to maintain the body in a relatively constant position with respect to the screen. Next, participants carried out a series of head movements and eye movements to calibrate the eye-tracker. Participants then drove a training session, followed by an optional break, and the driving test consisting of four sessions. The sessions, including the training session, lasted 278 s each. After each session, participants filled out the NASA-TLX questionnaire for measuring workload. 

The environment for all sessions consisted of a two-lane highway without on- and off-ramps. The highway contained several slight bends and a 270 degree left curve of 300 m radius. The highway had a total length of 7534 m and a lane width of 3.6 m. No other moving vehicles shared the road with the participant.

The participants were asked to drive in the right lane of the two-lane highway. The control of the car consisted of steering only; the participants did not use the pedals or gear lever. The car accelerated automatically until reaching a constant speed of 100 km/h (reached 17 s after the car was started).

In session 1, the road was empty. In session 2, the road was also empty, and the participants performed a letter detection task as secondary task. Participants had to verbalize equally sized (5 cm height) green colored (RGB 16%, 100%, 16%) letters appearing on the projected environment. The letters automatically disappeared 4 s after their appearance, and intervals between the letters varied between 3 and 6 s. The first letter appeared 6 s after the session was started. Thirty-two letters per session were projected at 25 different preprogrammed coordinates on the screen. 

Based on the positions of the participants’ eyes with respect to the screen as measured during the experiment, we estimated the horizontal and vertical peripheral eccentricities of the projected letters. The mean horizontal angle across participants between a line through the leftmost projected letter and the origin of gaze (i.e., the point between both eyes) and a line perpendicular to the screen through the origin of gaze was -6.4 degrees (SD = 1.4). For the rightmost projected letter this horizontal angle was 36.1 (SD = 2.3) degrees. The vertical mean angles of the lowest and highest projected letters were estimated at -2.9 (SD = 2.3) and 16.8 (SD = 2.7) degrees, respectively. Note that the nonzero standard deviations arise from individual differences in seating position and eye height.

In session 3, the participants had to evade nine static obstacles by changing to the left lane. The obstacles were positioned in the center of the right lane, and were three bicycles, three pairs of bicycles, and three cars. The participants encountered the obstacles at the following times in seconds: 19.7, 49.2, 61.7, 83.4, 121.8, 135.1, 226.0, 236.8, and 267.9 (corresponding distances in meters: 350, 1171, 1521, 2125, 3193, 3563, 6095, 6395, and 7262). 

In session 4, the participants had to both perform the letter task and evade the same nine obstacles as in session 3. The times and positions in which the letters and obstacles appeared in session 4 were identical to those in sessions 2 and 3, respectively. The actual letter characters were different from session 2. In the training session, the participants had to evade five static obstacles located at the positions corresponding to the 2nd, 4th, 6th, 8th, and 9th obstacles of sessions 3 and 4. The distance between the car and obstacle at which obstacles became perceivable (due to the finite resolution of the LCD projector) was estimated at 135 m.

### Demographic and driving habit data

The following items were selected from the 37-item questionnaire: (1) age, (2) gender, (3) worry about sight (1 = never, 7 = always), (4) medication use, (5) number of collisions involving damage in the past 36 months, as a driver, (6) number of kilometers driven in the past 12 months, (7) driving frequency in the past 12 months (1 = every day, 7 = never), and (8) cruise control experience (no or yes). These items were considered relevant for assessing the (non)equivalence of the glaucoma group versus the control group.

### Visual field measures

The monocular visual fields were merged into an integrated visual field (IVF) by determining the best monocular sensitivity for 52 corresponding positions around visual fixation. Furthermore, a measure of sensitivity loss was calculated for the monocular (better eye and worse eye) and integrated visual fields, based on a method reported by Szlyk et al. [9]. Sensitivity measures represented the percentage of 13 points (i.e., 52/4) falling below a 25 dB sensitivity threshold for the quadrants of the visual field (upper left, upper right, lower left, and lower right). The mean deviation (MD) per eye was recorded as well for each participant.

### Reaction time

Participants performed the Deary-Liewald reaction time task, consisting of a simple reaction time (SRT) and a choice reaction time (CRT) task [29]. Reaction time is associated with health and general cognitive ability [29], and studies suggest that similar neurodegenerative mechanisms may underlie both glaucoma and cognitive decline [8,30,31]. The reaction time tasks were included to verify whether glaucoma and control groups had equal ability on this elementary cognitive task. 

### Driving simulator

A fixed-base interactive driving simulator (Green Dino, Wageningen, The Netherlands) was used. The simulator cabin was equipped with the following components: steering wheel, ignition key, gear lever, single seat, and pedals. The feedback of the steering wheel was provided by a passive spring system. Surround sound was used to simulate wind, tires, and engine noise. The virtual environment, including dashboard and rear-view mirror, was projected onto the central screen (1.09 m X 0.77 m) by means of an LCD projector (NEC VT676) providing an image of 1024 x 768 pixels. With the exception of projected side mirrors, the lateral screen projections were not used in order to reduce driving simulator discomfort [32]. The total field of view (FOV) perceived by the participant was approximately 70 degrees horizontally and 50 degrees vertically, depending on the distance between the seat and the screen.

### Driving performance measures

The steering angle and vehicle position were recorded with a sampling frequency of 60 Hz. High frequency noise from the steering wheel angle was lowpass filtered (Butterworth, 2nd order) with two cutoff frequencies: 0.5 Hz and 2 Hz. The maximum frequency with which a human can steer is about 1 to 2 Hz [33]. Hence, 2 Hz is an appropriate cutoff frequency to remove sensor noise, whereas a cutoff frequency of 0.5 Hz filters out high-frequency steering inputs (e.g., fierce steering around obstacles) and focuses on the driver’s steering oscillations. 

The following measures were calculated per session:

• Steering activity (SA) (deg/s) was defined as the average speed of the steering wheel angle [34]. This metric was defined in two different ways: based on the steering angle filtered with high (SA-high; 2 Hz) and low (SA-low; 0.5 Hz) cutoff frequencies. SA-high and SA-low were calculated for obstacle periods (defined as sections starting 150 m before and ending 200 m after obstacles) and outside obstacle periods, regardless of whether the obstacles were actually present in that session. Steering activity can be seen as a measure of driving style, where low steering activity indicates smooth steering, and a high steering activity describes active steering.
• Standard deviation of lateral position (SDLP) (m) is a commonly used measure, describing the vehicle’s swerving on the road. A high SDLP value indicates imprecise lane keeping [35,36].
• Longitudinal distance to obstacle (LongDtO) (m), a measure of reaction with respect to the static obstacles, with a higher value indicating that the participant reacted earlier. LongDtO was defined as the longitudinal distance between the center of the obstacle and center of participant’s vehicle when the latter crossed the road center (i.e., the line between the two lanes). The mean of the nine obstacles was calculated.
• Lateral position to obstacle (LatDtO) (m), the lateral distance between the center of the obstacle and the center of the participant’s vehicle when passing the obstacle. The mean of the nine obstacles was calculated.
• A collision was defined as a lateral distance between the center of the participant’s vehicle and the center of an obstacle of less than 1.6 m (contact implied from the car’s dimensions) while driving alongside the obstacle. Note that physical impact would never occur because the participants’ car could move unimpeded through the obstacles.

### Letter task measures

The projector (EPSON EPN30) used for the letter stimuli was mounted on top of the projector used for projecting the virtual environment. Sessions 2 and 4 were recorded with a microphone that was unobtrusively fixed to the simulator’s cabin. Times of voice events were extracted automatically from the auditory signal. The reaction time was defined as the elapsed time between the letter appearance and the voice event. A letter was considered as detected if the participant called out any sound within the time in which the letter was projected until one second after its disappearance. Accordingly, the number of misses, and the average letter reaction time (LRT) per session were obtained. Spearman correlations between the letter measures (number of misses and reaction time) and the sensitivity-loss percentages were calculated for the glaucoma group. 

### Eye-scanning measures

A remote eye tracker (Smart Eye 5.9, Sweden) was used to measure eye gaze during driving. The eye tracker consisted of three Sony XC-HR50 cameras equipped with two infrared illuminators mounted below the virtual scenery of the driving simulator. Data was recorded with a sampling frequency of 60 Hz and was lowpass filtered (Butterworth, 2nd order) with a 20 Hz cutoff frequency. 

**Figure 1:**
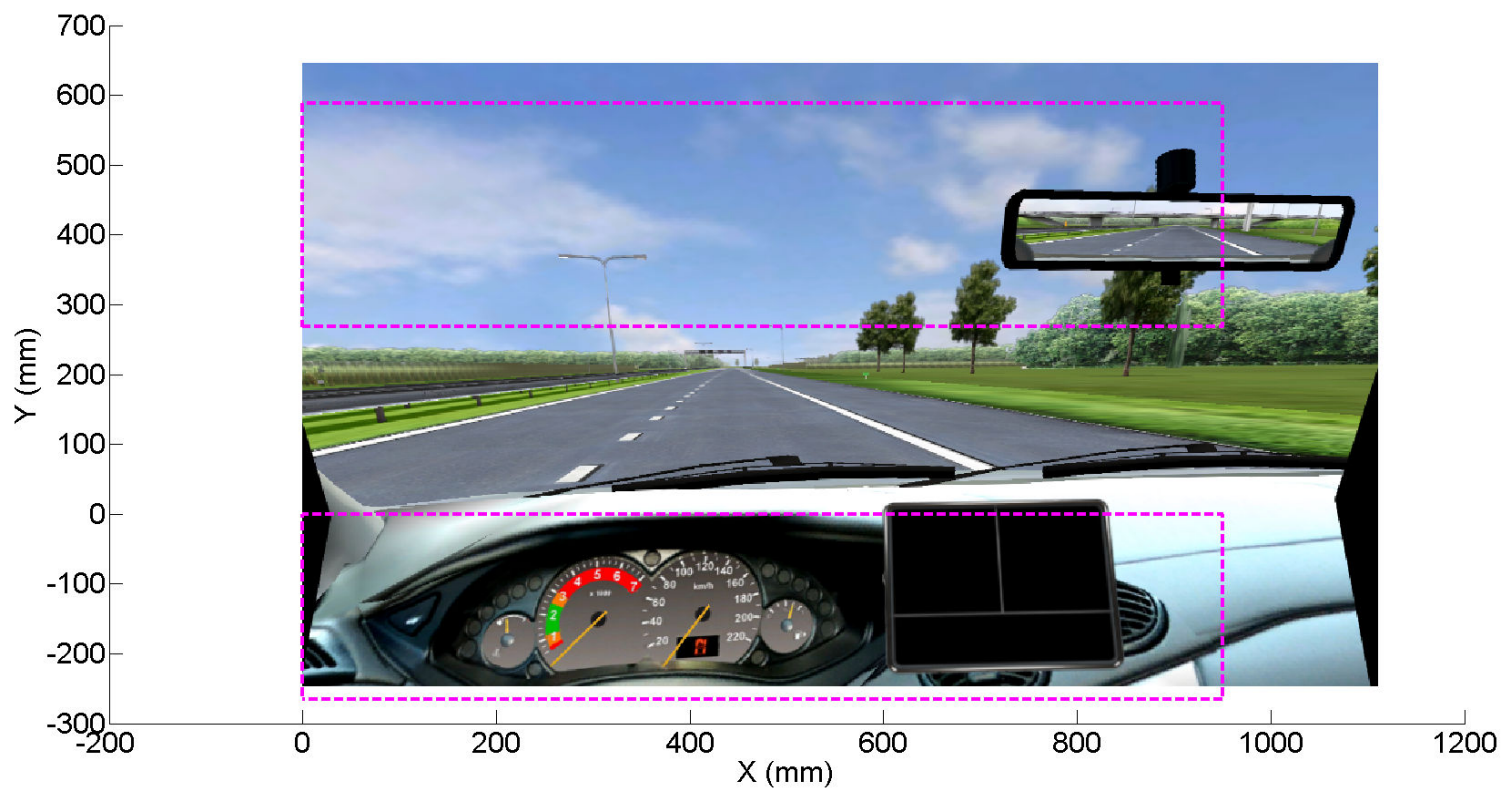
Illustration of top and bottom regions (inside dashed rectangles) used for calculating the eye-scanning measures.

The following eye-scanning measures were calculated per session:

• Percentage of time that gaze was directed at the screen’s top and bottom, as a measure of visual attention (see Figure 1 for an illustration).
• Number of fixations per second, a measure of the visual search strategy of participants [23]. Fixations were defined according to a dispersion-based method with a threshold of 3 degrees and a minimum fixation duration of 150 m [37,38].
• Mean saccade amplitude (mm), a measure of visual scanning, calculated as the mean distance between subsequent fixations [23,39].

### Self-reported workload

A Dutch version of the NASA-TLX [40], a common questionnaire in driving simulator research (e.g., [41,42]), was used to verify whether glaucoma patients and control participants experienced similar workload. The NASA TLX includes the following six items: mental demand, physical demand, temporal demand, performance, effort, and frustration. Scores were marked on a 21-tick bar ranging from 1 = very low to 21 = very high (1 = perfect and 21 = failure for the performance item). A total score was calculated by averaging the six items and expressing the results on a scale from 0% (lowest rating on all items) to 100% (highest rating on all items).

### Statistical tests

Group comparisons (glaucoma patients vs. control participants) were conducted by means of a two-sample t-test assuming that the two samples came from normal distributions with unknown and unequal variances (also known as Welch’s t-test). Differences between sessions were evaluated by means of a paired t-test. Associations between variables were evaluated using the Spearman correlation coefficient. Analyses were two-tailed and the α value (i.e., the false positive rate) was set at 0.05. 

No α correction for multiple testing was made, because our sample size was relatively small. Fielder et al. [43] explained that “it remains a statistical fact that measures that decrease α will often increase β”.Mudge et al. [44] argued that researchers should aim to minimize the combined probability (or cost) of Type I errors (i.e., false positives) and Type II errors (i.e., false negatives). Mudge et al. showed that when the sample size is smaller, a more liberal α value should be set to minimize the total probability of error. We argued that setting the α value below 0.05 would disproportionally increase β, the false negative rate. 

## Results

### Excluded data

The data from two glaucoma patients and two control participants was excluded from the analyses. These participants experienced simulator discomfort during the training session and were at that point withdrawn from the experiment. Accordingly, data from 35 participants were used for further analyses. 

Eye-scanning recordings may be inaccurate because of the system’s inability to detect facial features or pupils. This problem can be aggravated by reflections and physical obstructions of a participant’s glasses. The data from two participants were excluded after visual inspection of the video recordings from the eye-tracking system. Their data was removed because the eye-tracker measured erratic gaze patterns contradicting pupil movements. Data samples corresponding to 0.5 s before and after blinks, missing data values, and data segments of less than 1 s were removed. Eye-tracking data of participants was completely excluded, if one or more sessions had more than 60% removed data. In total, the data from 8 participants (7 patients, 1 control) was excluded from further eye-scanning analyses.

### Descriptive statistics

Table 1 shows descriptive statistics for both groups. There was no statistically significant difference for mileage, driving frequency, SRT, and CRT between the glaucoma group and the control group. Participants from the control group reported to be more experienced with cruise control, and were less likely to wear glasses during the experiment than glaucoma patients. Three participants (one patient and two control participants) had participated in a pilot test 7 months prior to this experiment. Two of the glaucoma patients reported to be under the influence of ophthalmic medication that may affect driving. 

**Table 1:** Means (standard deviations in parentheses) for participants’ characteristics, mean deviation of the visual field, and reaction times from the Deary-Liewald task.

**Measure**	**Control group**	**Glaucoma group**	
	**(n = 12)**	**(n = 23)**	**p**
Age (years)	65.7 (9.4)	65.1 (12.2)	0.877
Gender (% women)	25	35	0.560
Percentage of IVF points < 25 dB	1.9 (2.7)	42.4 (25.3)	1.1*10^-7^
Mean deviation OD	-0.09 (0.86)	-10.72 (8.98)	1.0*10^-5^
Mean deviation OS	-0.16 (1.16)	-13.99 (7.04)	2.3*10^-9^
Wore glasses during test (% of participants)	25	70	**0.012**
Single reaction time (s)	0.29 (0.05)	0.30 (0.04)	0.696
Choice reaction time (s)	0.52 (0.11)	0.54 (0.06)	0.494
Annual mileage (km)^1^	11,478 (9,080)	8,909 (8,694)	0.429
Driving frequency (1 = every day, 7 = never)^1^	2.08 (1.73)	2.78 (1.83)	0.277
Worry about sight (1 = never, 7 = always)^1^	1.33 (0.65)	2.43 (0.99)	4.3*10^-4^
Number of collisions in past 36 months^1^	0.17 (0.39)	0.35 (0.49)	0.242
Experience with cruise control (% of participants)^1^	67	26	**0.027**

^1^ Self-reported data; IVF = integrated visual field; dB = decibel; OD = oculus dexter (right eye); OS = oculus sinister (left eye).

### Driving performance of glaucoma group vs. control group

The results from the driving measures averaged over sessions are presented in Table 2. The glaucoma group displayed significantly higher steering activity than the control group on the following three metrics: SA-high (obstacle periods), SA-low (non-obstacle periods), and SA-low (obstacle periods). There were no statistically significant differences between the two groups for SA-high (non-obstacle periods), SDLP, LongDtO, and LatDtO.

**Table 2:** Means (standard deviations in parentheses) for participants’ driving measures.

**Measure**	**Control group**	**Glaucoma group**	**p**
	**(n = 12)**	**(n = 23)**	
Standard deviation of lateral position (SDLP), non-obstacle periods (S1-S4) (m)	0.43 (0.16)	0.37 (0.08)	0.262
Steering Activity-high, non-obstacle periods (S1-S4) (deg/s)	1.92 (0.50)	2.21 (0.65)	0.159
Steering Activity-high, obstacle periods (S3,S4) (deg/s)	3.00 (0.69)	3.86 (1.17)	**0.010**
Steering Activity-low, non-obstacle periods (S1-S4) (deg/s)	0.99 (0.14)	1.16 (0.18)	**0.004**
Steering Activity-low, obstacle periods (S3,S4) (deg/s)	1.70 (0.34)	2.27 (0.53)	5.0*10^-4^
Longitudinal distance to obstacle (LongDtO) (S3, S4) (m)	51 (12)	55 (11)	0.319
Lateral position to obstacle (LatDtO) (S3, S4) (m)	3.01 (0.35)	3.07 (0.28)	0.583

S3 = session 3; S4 = session 4; S1-S4 = sessions 1 to 4.

**Figure 2:**
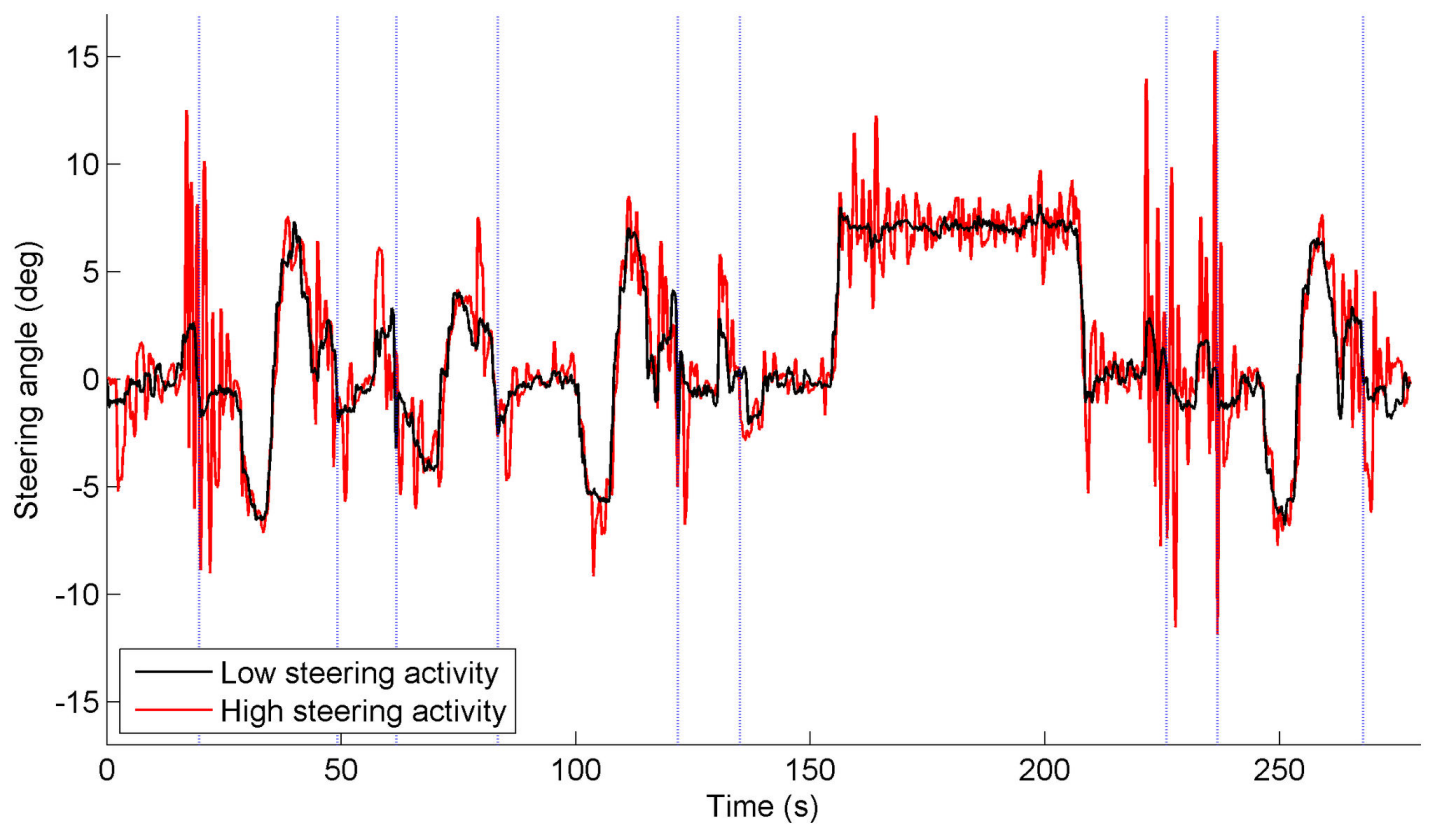
Steering angle versus time of two selected participants in session 4. One selected participant had very active steering, whereas the other had very smooth steering. The steering angle was filtered with a cutoff frequency of 2 Hz. Positive = steering to left, negative = steering to right. The dotted vertical lines indicate when the participant passed one of the nine static obstacles.

**Figure 3:**
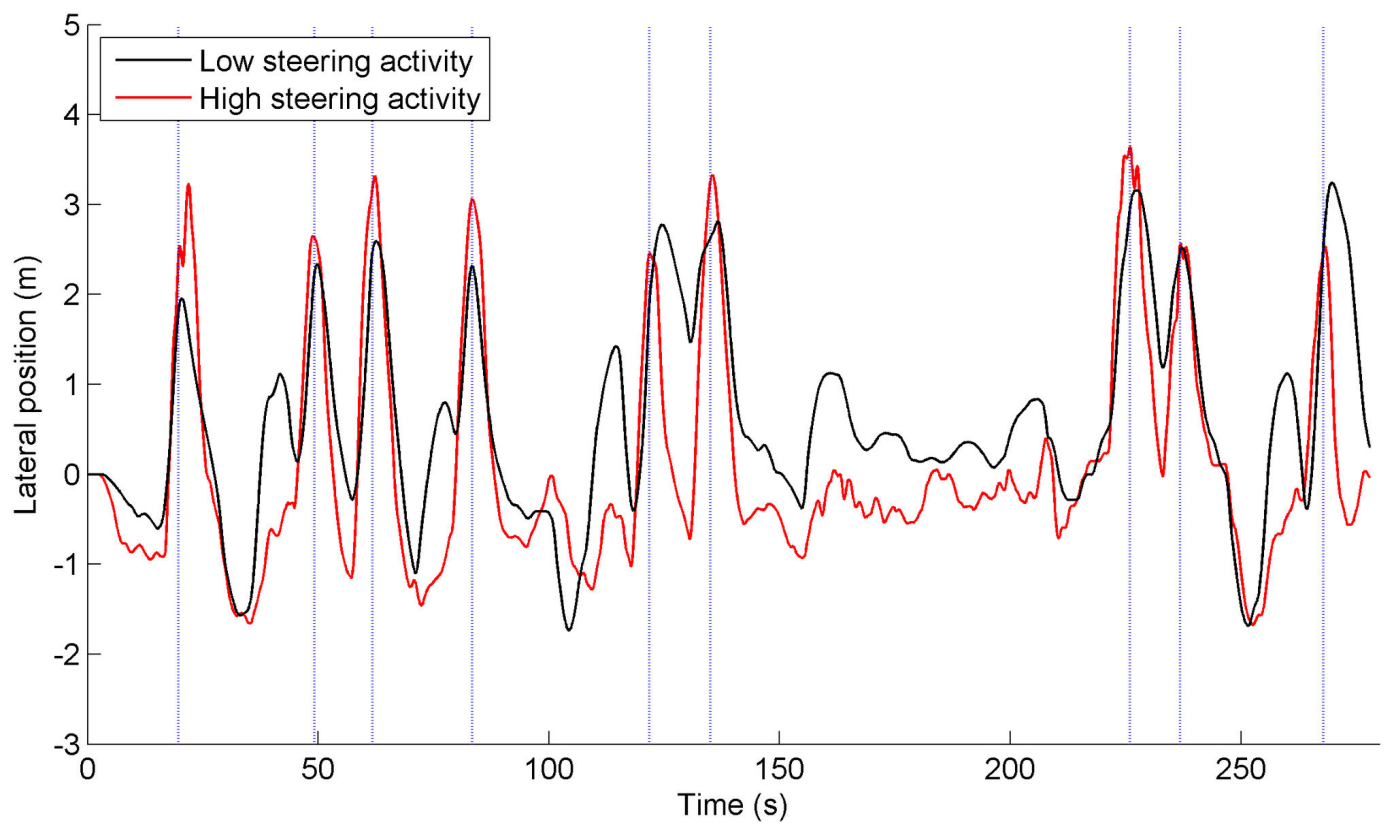
Lateral position versus time of two selected participants in session 4. One selected participant had very active steering, whereas the other had very smooth steering. Positive = lateral position to left with respect to right-lane center, negative = lateral position to right with respect to right-lane center. The dotted vertical lines indicate when the participant passed one of the nine static obstacles.

Figure 2 illustrates the difference between low and high steering activity. For illustrative purposes, the participant with the minimum and the participant with maximum value of SA-high in session 4 were selected. The corresponding SA-high values for these two participants (one control participant, and one glaucoma patient, respectively) were 1.28 deg/s and 2.70 deg/s for the non-obstacle periods, and 1.54 deg/s and 6.91 deg/s for the obstacle periods. Figure 3 illustrates the lateral position for the same two participants in session 4. The corresponding SDLP values for the non-obstacle periods were 0.73 m for the participant with low steering activity and 0.46 m for the participant with high steering activity. It can be seen in Figure 3 that the person with low steering activity took longer to go back to the right lane after avoiding the obstacle, had larger lane center errors, and had smaller lateral margins around obstacles, than the participant with high steering activity.

The analyses indicated that there were no collisions for any of the participants. Within the group of 23 glaucoma patients, none of the correlations between the percentage of depressed IVF points and the driving performance measures listed in Table 2 reached statistical significance (p > 0.2 for all correlations).

### Letter task performance of glaucoma group vs. control group

The glaucoma group missed significantly more letters and had a significantly longer letter reaction time than the control group (Table 3). Within the group of 23 glaucoma patients, people with a larger percentage of depressed IVF points had longer reaction times (ρ = 0.62, p = 0.001) and a higher number of letter misses (ρ = 0.67, p = 5.0*10^-4^).

**Table 3:** Means (standard deviations in parentheses) for participants’ letter misses and letter reaction times.

**Measure**	**Control group**	**Glaucoma group**	**p**
	**(n = 12)**	**(n = 23)**	
Letter Reaction Time (LRT) (S2, S4) (s)	1.06 (0.20)	1.48 (0.26)	8.9*10^-6^
Number of letter misses (S2, S4)	1.83 (0.91)	6.13 (5.22)	7.8*10^-4^

S2 = session 2; S4 = session 4.

### Eye-scanning of glaucoma group vs. control group

**Figure 4:**
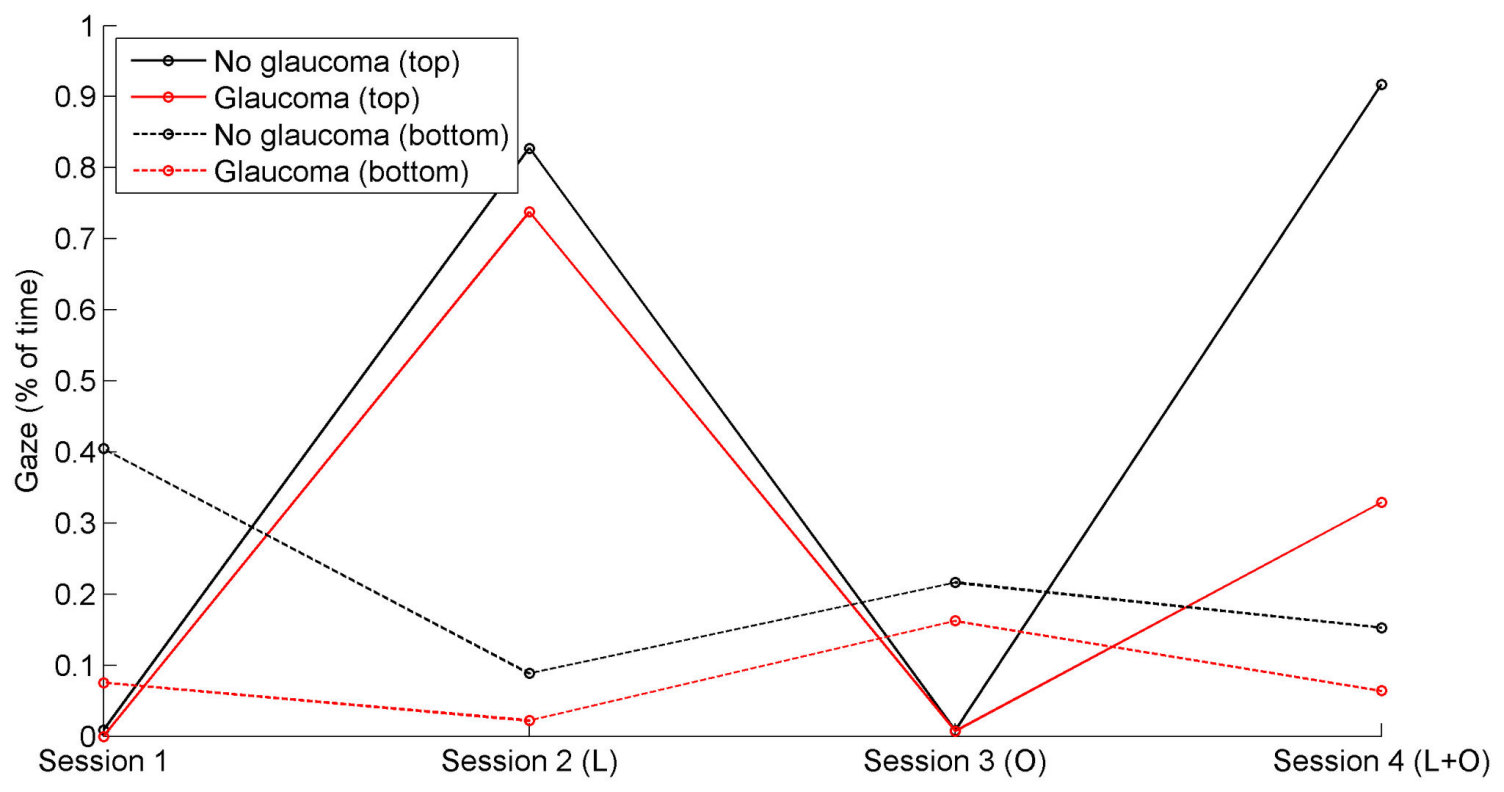
Median of percentage of time that gaze was directed at the top and bottom regions of the screen, per session (L = letter task, O = obstacle avoidance task).

Table 4 shows that participants from both groups hardly looked at the top and bottom regions of the screen. Table 4 further shows that there were no statistically significant differences between the glaucoma group and the control group for the eye-scanning measures. Figure 4 illustrates the median percentage of time per session that gaze was directed at the upper and lower regions of the screen. There were no statistically significant differences between the two groups for any session.

**Table 4:** Means (standard deviations in parentheses) for participants’ eye-scanning measures.

**Measure**	**Control group**	**Glaucoma group**	**p**
	**(n = 11)**	**(n = 16)**	
Gaze directed at top (S1-S4) (% of time)	0.70 (0.65)	0.39 (0.40)	0.181
Gaze directed at bottom (S1-S4) (% of time)	0.47 (0.65)	0.56 (1.37)	0.815
Number of fixations per second (S1-S4)	0.59 (0.32)	0.58 (0.28)	0.942
Mean saccade amplitude (S1-S4) (mm)	278 (91)	300 (90)	0.569

S1-S4 = sessions 1 to 4.

An additional exploratory analysis showed that participants with a larger percentage of IVF points depressed below the 25 dB sensitivity threshold tended to gaze less to the top (ρ = -0.41, p = 0.033) and bottom parts (ρ = -0.35, p = 0.076) of the screen in sessions 1 to 4 combined (n = 27). Similarly, participants with a lower mean deviation (dB) were less likely to gaze to the top and bottom parts of the screen (ρ = 0.41, p = 0.033 for the top part; ρ = 0.43, p = 0.025 for the bottom part; MD of both eyes averaged).

**Figure 5:**
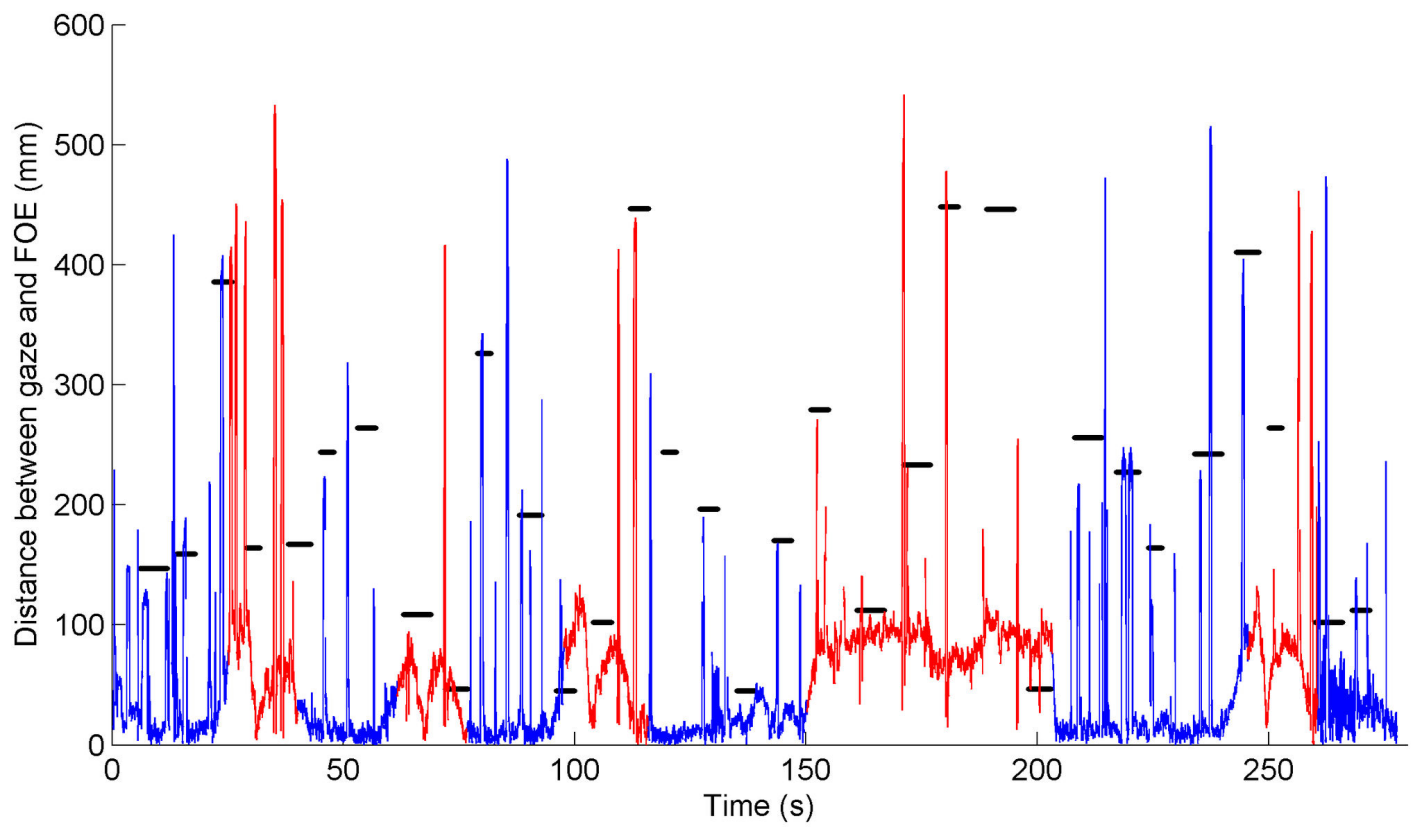
Distance between gaze and focus of expansion (FOE; static point on the screen) versus time, for one typical participant during session 2 (from the control group). The line is red when driving on a curved road segment; the line is blue when driving on a straight road segment. Distances from the projected letters to the FOE are indicated by horizontal black lines. Distances from the FOE to the rear-view mirror, speedometer, and side mirrors are 450 mm, 300 mm, and 600 mm, respectively.

Figure 5 illustrates the eye-scanning behavior of a typical participant during session 2. The participant directed his gaze mostly to the focus of expansion (i.e., the point on the horizon from which, when being in forward motion, the optic flow seems to emanate), directed attention to the letters shortly after they became visible, and immediately directed attention back to the center region. This participant did not display noticeable active visual search in between letter projections; the percentage of session time that this participant spent looking toward the top and bottom regions was 0.45% and 0.00%, respectively. 

### Self-reported workload of glaucoma group vs. control group

**Figure 6:**
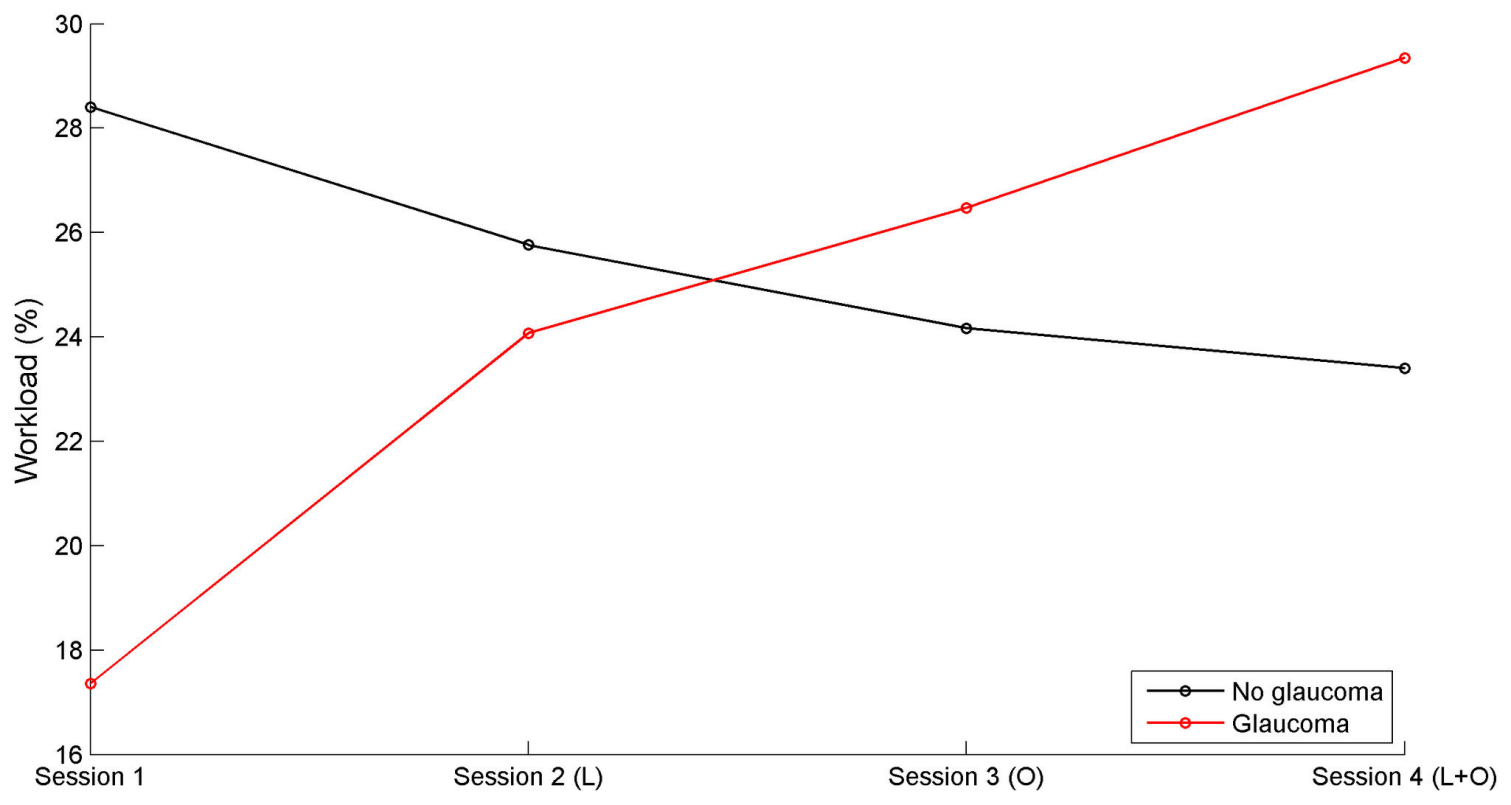
Mean self-reported workload per session (L = letter task, O = obstacle avoidance task).

There was no statistically significant difference between the control group and the glaucoma group for the mean self-reported workload across the four sessions (p = 0.894). The mean workload score for the control group and glaucoma group was 25% (SD = 18%) and 24% (SD = 15%), respectively. Figure 6 shows the mean workload reported by glaucoma patients and control participants per session. For the control group, there was a non-significant decrease of workload for session 1 versus session 4 (p = 0.233), whereas for the patient group the workload percentage significantly increased (p = 1.3*10^-4^).

### Correlations between sensitivity loss and letter task performance

The correlation matrix between sensitivity loss and letter measures is reported in Table 5. Quadrants from the upper visual field were strongly correlated with letter misses and letter reaction times (LRT). The quadrants in the lower visual field yielded relatively weak correlations with the letter measures. The IVF and better-eye VFs showed stronger correlations with letter performance than the worse-eye VF.

**Table 5:** Spearman correlation coefficients between glaucoma patients’ (n = 23) visual field loss (percentage of points below 25 dB sensitivity threshold per quadrant of the visual field), and number of letter misses and letter reaction time.

**VF location**	**VF type**	**Letter misses**	**LRT**
Upper-left	IVF	0.842	0.574
Upper-right	IVF	0.791	0.451
Lower-left	IVF	0.237	0.333
Lower-right	IVF	0.216	0.308
Upper-left	Worse eye	0.501	0.263
Upper-right	Worse eye	0.481	0.231
Lower-left	Worse eye	-0.061	0.156
Lower-right	Worse eye	-0.068	0.211
Upper-left	Better eye	0.832	0.631
Upper-right	Better eye	0.748	0.504
Lower-left	Better eye	0.302	0.344
Lower-right	Better eye	0.297	0.378

p < 0.05 for ρ ≥ 0.42; LRT = letter reaction time; VF = visual field.

**Figure 7:**
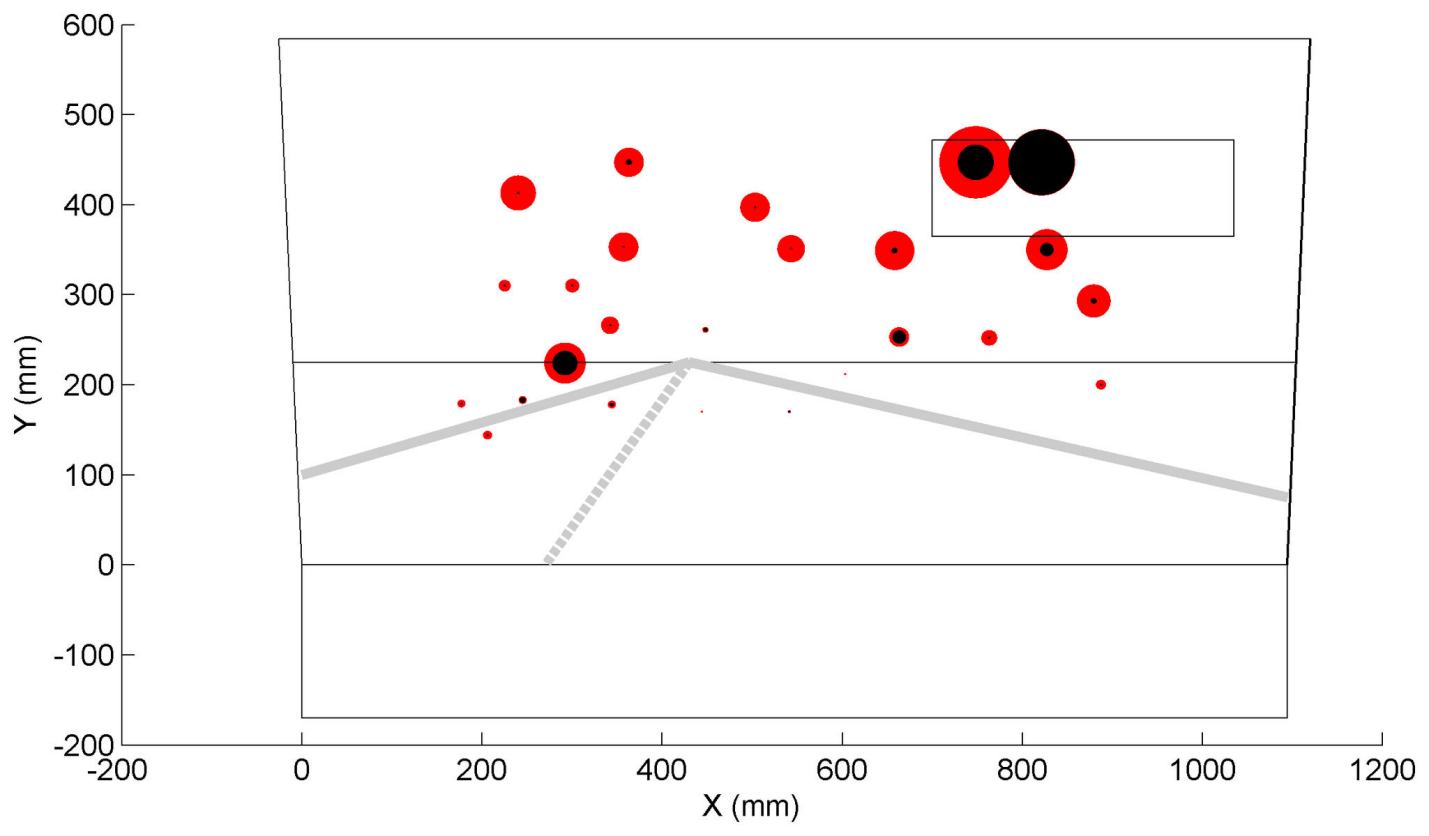
Mean number of letter misses for each projected letter position (red = patients, black = control participants); the radius of the circle linearly corresponds to the mean number of letter misses. Patients’ mean number of missed letters ranged between 0% (on the road) and 78% (rear-view mirror).

**Figure 8:**
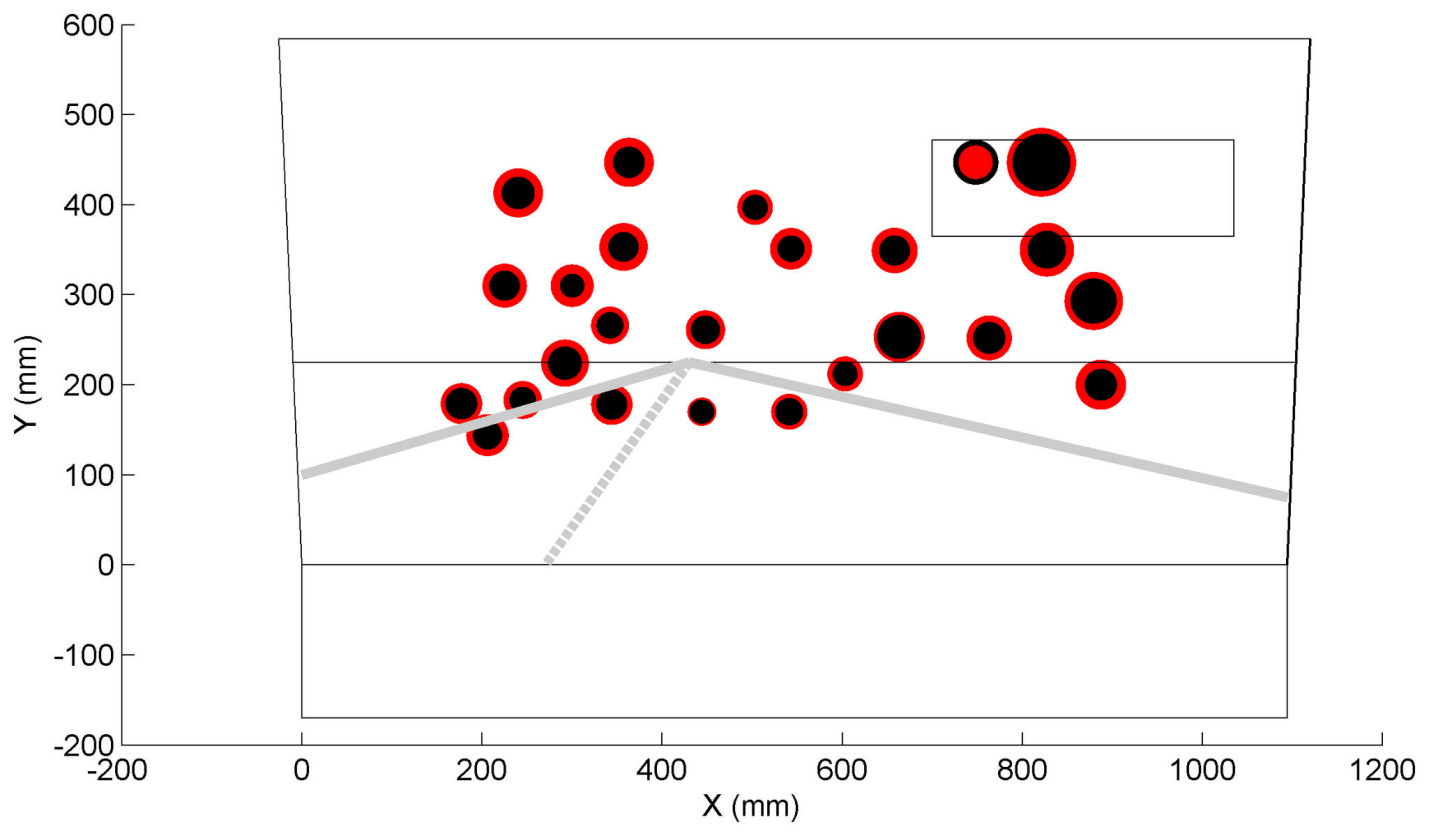
Mean letter reaction time for each projected letter position (red = patients, black = control participants); the radius of the circle linearly corresponds to the mean reaction time. Patients’ mean reaction times ranged between 0.98 (on the road) and 2.48 s (rear-view mirror).

Figures 7 and 8 show the positions on the simulator’s screen where letters were prone to be missed by participants and positions where participants showed higher reaction times, respectively. Most missed letters were those projected in the peripheral regions whereas the most successfully verbalized letters were those confined to the road region. 

## Discussion

The aim of this study was to evaluate differences in driving and visual detection performance between patients with mild to severe glaucoma and control participants without glaucoma. Eye-scanning behavior was evaluated considering that it may represent a compensation technique for visual field loss. 

Glaucoma patients performed indistinguishably from control participants on evading obstacles and keeping the car centered in the lane. However, the steering activity was higher for glaucoma patients than for controls. According to the optimal control theory of manned-vehicle systems, a human who performs a continuous tracking task can emphasize a target criterion, such as minimizing error with respect to a target or minimizing control effort [45]. The present results suggest that the patients gave more attention to minimizing lane keeping error (i.e., keeping the car centered in the lane), while the control group emphasized steering smoothness. These results are in line with Szlyk et al. [17] who found that glaucoma patients reacted faster than control participants after being presented with a stop sign. Szlyk et al. attributed this finding to patients’ hypervigilance, as this group might have been more concerned than control subjects that their driving performance was being evaluated. However, our results may also be interpreted as the effect of reduced visual field in glaucoma patients. The patients may have missed relevant optical cues that are used for perception of distance and heading, and accordingly, the increased steering activity of patients may suggest that they had more difficulty performing the driving task. 

Participants directed their gaze mostly to the focus of expansion, and no differences in eye-scanning behavior were detected between the two groups. In fact, our results indicated that participants with more severe visual field defects tended to look less to the upper and lower parts of the screen. This finding contradicts the idea that glaucoma patients compensate for their visual field loss by displaying increased visual search while driving, see 23 for a hazard perception study. During a classical hazard-perception task, participants are observers of a scenario, whereas in our driving simulator, participants had to control a vehicle themselves. Participants in a driving simulator may not be inclined to look away from the road center, as doing so imposes a risk of collision or loss of control of the vehicle. It should be noted however, that our sample size, and therefore statistical power, was small. 

The patient group missed substantially more letters than the control group. The result is consistent with Szlyk et al. [9] who reported that compensation mechanisms for patients with severe glaucoma are not effective for unpredictable events arising from the periphery. Our result is also in agreement with glaucoma patients’ deficiency in detecting unexpected objects (e.g., pedestrians) in real driving as found by Haymes et al. [5], and with patients’ impaired performance on a peripheral detection task as observed in a driving simulator study by Rosen et al. [8]. Glaucoma patients’ difficulty with aspects of car driving that involve reacting to unexpected events may explain their elevated motor vehicle collision involvement rates. It was interesting that both groups were prone to missing letters projected inside the rear-view mirror. This was probably due to the mirror’s delineation causing low letter conspicuity in comparison to letters projected on a relatively uniform sky. 

Our results showed that participants directed their gaze mostly toward the point of expansion. No letters were projected substantially below this region, which may explain why letter detection performance was strongly correlated with the participants’ upper visual field loss. Defects in the integrated and better-eye visual fields were more strongly correlated with letter detection performance than worse-eye metrics. This result is in line with studies showing that the IVF effectively approximates the binocular visual field and is a valid predictor of task performance [46,47], and with Saunders et al. [48] showing that the better eye visual field is a strong marker of legal fitness to drive. Note, however, that the integrated monocular visual field may be different than actual obstacle perception in the far temporal peripheries. In our study, 8 of the 32 letters were projected outside the IVF margin of 21 degrees (i.e., the 8 rightmost letters). It is further noted that the percentage of depressed points for the IVF and the better eye were strongly correlated (ρ = 0.96, n = 35), which explains why their correlations with letter misses were highly similar (cf. Table 5). 

The TLX questionnaire revealed no difference in subjective workload between the two groups. However, the progressive addition of tasks yielded an increase of workload in glaucoma patients, while no increase was observed in the control group. A factor which may explain the observed increase in reported workload among glaucoma patients is that the patients may have had different expectations toward the test than the control participants. It is possible that patients expected a challenging task given their visual impairment, and therefore rated session 1 as extraordinarily easy. As soon as more tasks (obstacle avoidance, letter detection) were added, the situation became more demanding for them and workload was rated as higher.

A driving simulator study by Engström et al. [49] found that increasing cognitive task demands (by means of the Auditory Continuous Memory Task) resulted in increased gaze concentration towards the road center, a phenomenon more commonly known as tunnel vision or cognitive tunneling. The increase of self-reported workload among glaucoma patients may be a factor explaining why they were more likely to miss peripherally projected letters than control participants. 

The control group reported to be more experienced with cruise control than the glaucoma group. Cruise control experience might have influenced our results, considering that driving speed was automatically controlled in our experiment. Future research may have to consider cruise control experience as an inclusion criterion. Generally, participants’ computer skills and experience with technological novelties may need to be controlled in future research.

Previous driving simulator studies [17,22] have shown that drivers compensate for their degraded visual ability by reducing their driving speed. In our study, the automated longitudinal control of the vehicle provided a highly controlled and uniform environment for all participants (i.e., equal task demands, same spatial-temporal appearance of objects and letters). Our approach prevented compensation by means of speed. The use of speed as a compensatory mechanism among glaucoma patients will have to be studied in future research.

Driving simulators are able to provide performance metrics that are predictive of real driving (e.g., [50]). However, driving simulators offer a limited degree of fidelity, and can cause simulator sickness in some participants. Some types of visual information, such as stereopsis, glare, and accommodation distance, were not provided by our simulator, nor did our simulator provide vestibular motion feedback. Furthermore, although the projected letters were useful to test participants’ reactions to unexpected events, letters do not occur in real-world driving environments. Future research could investigate glaucoma patients’ detection of naturally occurring events, by means of a driving simulator or an on-road test. Note, however, that on-road tests are known to pose serious challenges regarding experimental control and sensor data quality [51].

It should be considered that the driving sessions comprised less than 20 min of total driving time, while normal on-road driving may involve hours of sustained attention. Future research could explore the effects of fatigue during longer testing periods.

Finally, we note that our sample size was modest, thereby risking false positive and false negative findings [52]. It is recommended to carry out a replication study with a larger sample size, particularly for verifying the findings that are at the borderline of statistical significance.

In conclusion, our driving simulator study showed that glaucoma patients and control participants had statistically indistinguishable performance in lane keeping and obstacle avoidance. Glaucoma patients moved their steering wheel more actively, and had impaired performance on a visual detection task as compared to control participants. Finally, our eye-tracking data suggests that glaucoma patients did not apply a visual compensation mechanism to compensate for their visual field loss.

## Supporting Information

Video S1 **The instruction video shown to the participants.** In the actual experiment, the side projections were not used.
(AVI)

## References

[1] Resnikoff S, Pascolini D, Etya'ale D, Kocur I, Pararajasegaram R et al. (2004) Global data on visual impairment in the year 2002. Bull World Health Organ 82: 844-851. PubMed: 15640920. 15640920 PMC2623053

[2] Quigley HA, Broman AT (2006) The number of people with glaucoma worldwide in 2010 and 2020. Br J Ophthalmol 90: 262-267. doi:10.1136/bjo.2005.081224. PubMed: 16488940. 16488940 PMC1856963

[3] McGwin G, Mays A, Joiner W, DeCarlo DK, McNeal S et al. (2004) Is glaucoma associated with motor vehicle collision involvement and driving avoidance? Invest Ophthalmol Vis Sci 45: 3934-3939. doi:10.1167/iovs.04-0524. PubMed: 15505039. 15505039

[4] Ramulu PY, West SK, Munoz B, Jampel HD, Friedman DS (2009) Driving cessation and driving limitation in glaucoma: the Salisbury Eye Evaluation Project. Ophthalmology 116: 1846-1853. doi:10.1016/j.ophtha.2009.03.033. PubMed: 19592110. 19592110 PMC2757455

[5] Haymes SA, LeBlanc RP, Nicolela MT, Chiasson LA, Chauhan BC (2007) Risk of falls and motor vehicle collisions in glaucoma. Invest Ophthalmol Vis Sci 48: 1149-1155. doi:10.1167/iovs.06-0886. PubMed: 17325158. 17325158

[6] Hu PS, Trumble DA, Foley DJ, Eberhard JW, Wallace RB (1998) Crash risks of older drivers: a panel data analysis. Accid Anal Prev 30: 569-581. doi:10.1016/S0001-4575(98)00019-0. PubMed: 9678211. 9678211

[7] McGwin G, Xie A, Mays A, Joiner W, DeCarlo DK et al. (2005) Visual field defects and the risk of motor vehicle collisions among patients with glaucoma. Invest Ophthalmol Vis Sci 46: 4437-4441. doi:10.1167/iovs.05-0750. PubMed: 16303931. 16303931

[8] Rosen P, Akinwuntan A, Wachtel J, Boer E, Weinreb R et al. (2011) Driver fitness in patients with cognitive impairment and glaucoma. Proc Int Driv Symp Hum Factors Driv Assess Train Veh Des. pp. pp. 233-240

[9] Szlyk JP, Mahler CL, Seiple W, Edward DP, Wilensky JT (2005) Driving performance of glaucoma patients correlates with peripheral visual field loss. J Glaucoma 14: 145-150. doi:10.1097/01.ijg.0000151686.89162.28. PubMed: 15741817. 15741817

[10] Haymes SA, LeBlanc RP, Nicolela MT, Chiasson LA, Chauhan BC (2008) Glaucoma and on-road driving performance. Invest Ophthalmol Vis Sci 49: 3035-3041. doi:10.1167/iovs.07-1609. PubMed: 18326696. 18326696

[11] Bowers A, Peli E, Elgin J, McGwin G Jr, Owsley C (2005) On-road driving with moderate visual field loss. Optom Vis Sci 82: 657-667. doi:10.1097/01.opx.0000175558.33268.b5. PubMed: 16127330. 16127330

[12] Medeiros FA, Weinreb RN, Boer ER, Rosen PN (2012) Driving simulation as a performance-based test of visual impairment in glaucoma. J Glaucoma 21: 221-227. doi:10.1097/IJG.0b013e3182071832. PubMed: 21467952. 21467952 PMC3804259

[13] Rizzo M, Jermeland J, Severson J (2002) Instrumented vehicles and driving simulators. Gerontechnology 1: 291-296.

[14] Akinwuntan AE, DeWeerdt W, Feys H, Baten G, Arno P et al. (2003) Reliability of a road test after stroke. Arch Phys Med Rehabil 84: 1792-1796. doi:10.1016/S0003-9993(03)00767-6. PubMed: 14669185. 14669185

[15] Baughan C, Simpson H (2002). Graduated Licensing: A review of some current systems. TRL Research Report 529 UK: Transport Research Laboratory.

[16] Hunt LA, Murphy CF, Carr D, Duchek JM, Buckles V et al. (1997) Environmental cueing may affect performance on a road test for drivers with dementia of the Alzheimer type. Alzheimer Dis Assoc Disord 11: 13-16. doi:10.1097/00002093-199700112-00003. PubMed: 9194962. 9194962

[17] Szlyk JP, Taglia DP, Paliga J, Edward DP, Wilensky JT (2002) Driving performance in patients with mild to moderate glaucomatous clinical vision changes. J Rehabil Res Dev 39: 467-482. PubMed: 17638144. 17638144

[18] Bowers AR, Mandel AJ, Goldstein RB, Peli E (2009) Driving with hemianopia, I: Detection performance in a driving simulator. Invest Ophthalmol Vis Sci 50: 5137-5147. doi:10.1167/iovs.09-3799. PubMed: 19608541. 19608541 PMC2783572

[19] Kasper EF, Haworth LA, Szoboszlay ZP, King RD, Halmos ZL (1997) Effects of in-flight field-of-view restriction on rotorcraft pilot head movement. Proc SPIE: 34-45.

[20] Gallimore JJ, Brannon NG, Patterson FR (1998) The effects of field-of-view on pilot head movement during low level flight. Proc SPIE: 6-10.

[21] Szoboszlay Z, Haworth L, Reynolds T, Lee A, Halmos Z (1995) Effect of field-ofview restriction on rotocraft pilot workload and performance: preliminary results. Proc SPIE: 142-153.

[22] Coeckelbergh TR, Brouwer WH, Cornelissen FW, Van Wolffelaar P, Kooijman AC (2002) The effect of visual field defects on driving performance: a driving simulator study. Arch Ophthalmol 120: 1509-1516. doi:10.1001/archopht.120.11.1509. PubMed: 12427065. 12427065

[23] Crabb DP, Smith ND, Rauscher FG, Chisholm CM, Barbur JL et al. (2010) Exploring eye movements in patients with glaucoma when viewing a driving scene. PLOS ONE 5: e9710. doi:10.1371/journal.pone.0009710. PubMed: 20300522. 20300522 PMC2838788

[24] Lockhart J, Boyle LN, Wilkinson M (2009) Driving With Visual Field Loss: An Exploratory Simulation Study. DOT HS 811 062. National Highway Traffic Safety Administration.

[25] Owsley C, Stalvey B, Wells J, Sloane ME (1999) Older drivers and cataract: driving habits and crash risk. J Gerontol A Biol Sci Med Sci 54: M203-M211. doi:10.1093/gerona/54.4.M203. PubMed: 10219012. 10219012

[26] Parrish RK (1996) Visual impairment, visual functioning, and quality of life assessments in patients with glaucoma. Trans Am Ophthalmol Soc 94: 919–1028. PubMed: 8981717. 8981717 PMC1312116

[27] De Winter JC (2013) Predicting self-reported violations among novice license drivers using pre-license simulator measures. Accid Anal Prev 52: 71-79. doi:10.1016/j.aap.2012.12.018. PubMed: 23298709. 23298709

[28] Reason J, Manstead A, Stradling S, Baxter J, Campbell K (1990) Errors and violations on the roads: a real distinction? Ergonomics 33: 1315-1332. doi:10.1080/00140139008925335. PubMed: 20073122. 20073122

[29] Deary IJ, Liewald D, Nissan J (2011) A free, easy-to-use, computer-based simple and four-choice reaction time programme: the Deary-Liewald reaction time task. Behav Res Methods 43: 258-268. doi:10.3758/s13428-010-0024-1. PubMed: 21287123. 21287123

[30] Kirby E, Bandelow S, Hogervorst E (2010) Visual impairment in Alzheimer's disease: a critical review. J Alzheimers Dis 21: 15-34. PubMed: 20182034. 20182034 10.3233/JAD-2010-080785

[31] McKinnon SJ (2003) Glaucoma: ocular Alzheimer’s disease. Front Biosci 8: s1140-s1156. doi:10.2741/1172. PubMed: 12957857. 12957857

[32] Lin J-W, Duh HB, Parker DE, Abi-Rached H, Furness TA (2002) Effects of field of view on presence, enjoyment, memory, and simulator sickness in a virtual environment. Proc IEEE Virtual Real Conf. pp. pp. 164-171

[33] McRuer DT, Jex HR (1967) A review of quasi-linear pilot models. IEEE Trans Hum Factors Electron: 231-249.

[34] Saffarian M, Happee R, De Winter J (2012) Why do drivers maintain short headways in fog? A driving-simulator study evaluating feeling of risk and lateral control during automated and manual car following. Ergonomics 55: 971-985. doi:10.1080/00140139.2012.691993. PubMed: 22804738. 22804738

[35] Brookhuis KA, Waard DD, Fairclough SH (2003) Criteria for driver impairment. Ergonomics 46: 433-445. doi:10.1080/001401302/1000039556. PubMed: 12745694. 12745694

[36] Dijksterhuis C, Brookhuis KA, De Waard D (2011) Effects of steering demand on lane keeping behaviour, self-reports, and physiology. A simulator study. Accid Anal Prev 43: 1074-1081. doi:10.1016/j.aap.2010.12.014. PubMed: 21376904. 21376904

[37] Salvucci DD, Goldberg JH (2000) Identifying fixations and saccades in eye-tracking protocols. Proc Symp Eye Tracking Res App. pp. 71-78.

[38] Hornof AJ, Halverson T (2002) Cleaning up systematic error in eye-tracking data by using required fixation locations. Behav Res Methods Instrum Comput 34: 592-604. doi:10.3758/BF03195487. PubMed: 12564562. 12564562

[39] Underwood G, Crundall D, Chapman P (2011) Driving simulator validation with hazard perception. Transp Res Part F Traffic Psychol Behav 14: 435-446. doi:10.1016/j.trf.2011.04.008.

[40] Hart SG (2006) NASA-task load index (NASA-TLX); 20 years later. Proc Hum Fact Ergon Soc Annu Meet. pp. pp. 904-908

[41] Horberry T, Anderson J, Regan MA, Triggs TJ, Brown J (2006) Driver distraction: the effects of concurrent in-vehicle tasks, road environment complexity and age on driving performance. Accid Anal Prev 38: 185-191. doi:10.1016/j.aap.2005.09.007. PubMed: 16226211. 16226211

[42] de Winter JC, Mulder M, Van Paassen MM, Abbink DA, Wieringa PA (2008) A two-dimensional weighting function for a driver assistance system. IEEE Trans Syst Man Cybern B Cybern 38: 189-195. doi:10.1109/TSMCB.2007.908860. PubMed: 18270090. 18270090

[43] Fiedler K, Kutzner F, Krueger JI (2012) The long way from α-error control to validity proper: Problems with a short-sighted false-positive debate. Perspect Psychol Sci 7: 661-669. doi:10.1177/1745691612462587. 26168128

[44] Mudge JF, Baker LF, Edge CB, Houlahan JE (2012) Setting an optimal α that minimizes errors in null hypothesis significance tests. PLOS ONE 7: e32734. doi:10.1371/journal.pone.0032734. PubMed: 22389720. 22389720 PMC3289673

[45] Jagacinski RJ, Flach J (2011) Control theory for humans: Quantitative approaches to modeling performance. Taylor & Francis.

[46] Crabb DP, Fitzke FW, Hitchings RA, Viswanathan AC (2004) A practical approach to measuring the visual field component of fitness to drive. Br J Ophthalmol 88: 1191-1196. doi:10.1136/bjo.2003.035949. PubMed: 15317714. 15317714 PMC1772325

[47] Owen VM, Crabb DP, White ET, Viswanathan AC, Garway-Heath DF et al. (2008) Glaucoma and fitness to drive: using binocular visual fields to predict a milestone to blindness. Invest Ophthalmol Vis Sci 49: 2449-2455. doi:10.1167/iovs.07-0877. PubMed: 18515585. 18515585

[48] Saunders LJ, Russell RA, Crabb DP (2012) Practical landmarks for visual field disability in glaucoma. Br J Ophthalmol 96: 1185-1189. doi:10.1136/bjophthalmol-2012-301827. PubMed: 22797319. 22797319

[49] Engström J, Johansson E, Östlund J (2005) Effects of visual and cognitive load in real and simulated motorway driving. Transp Res Part F Traffic Psychol Behav 8: 97-120. doi:10.1016/j.trf.2005.04.012.

[50] Lee HC, Cameron D, Lee AH (2003) Assessing the driving performance of older adult drivers: on-road versus simulated driving. Accid Anal Prev 35: 797-803. doi:10.1016/S0001-4575(02)00083-0. PubMed: 12850081. 12850081

[51] Santos J, Merat N, Mouta S, Brookhuis K, De Waard D (2005) The interaction between driving and in-vehicle information systems: Comparison of results from laboratory, simulator and real-world studies. Transp Res Part F Traffic Psychol Behav 8: 135-146. doi:10.1016/j.trf.2005.04.001.

[52] Ioannidis JP (2005) Why most published research findings are false. PLOS Med 2: e124. doi:10.1371/journal.pmed.0020124. PubMed: 16060722. 16060722 PMC1182327

